# StressLife: A Short-Time Approach for the Determination of a Trend S-N Curve in and beyond the HCF Regime for the Steels 20MnMoNi5-5 and SAE 1045

**DOI:** 10.3390/ma16113914

**Published:** 2023-05-23

**Authors:** Fabian Weber, Janina Koziol, Peter Starke

**Affiliations:** 1 Department of Materials Sciences and Materials Testing (WWHK), Institude QM^3^, University of Applied Sciences, Schoenstraße 11, 67659 Kaiserslautern, Germany; janina.koziol@hs-kl.de (J.K.); peter.starke@hs-kl.de (P.S.); 2 Faculty of Natural Sciences and Technology, Saarland University, 66123 Saarbrücken, Germany

**Keywords:** fatigue, short-time procedure, load increase test, non-destructive testing, 20MnMoNi5-5

## Abstract

Within the scope of this research, a new short-time procedure designated as $StressLife_{HCF}$ was developed. Through a combination of classic fatigue testing and non-destructive monitoring of the material response due to cyclic loading, a process-oriented fatigue life determination can be carried out. A total of two load increases and two constant amplitude tests are required for this procedure. By using data from non-destructive measurements, the parameters of the elastic approach according to Basquin and the plastic approach according to Manson–Coffin were determined and combined within the $StressLife_{HCF}$ calculation. Furthermore, two additional variations of the $StressLife_{HCF}$ method were developed in order to be able to accurately describe the S-N curve over a wider range. The main focus of this research was 20MnMoNi5-5 steel, which is a ferritic-bainitic steel (1.6310). This steel is widely used for spraylines in German nuclear power plants. In order to validate the findings, tests were also performed on an SAE 1045 steel (1.1191).

## 1. Introduction

For components, structures, and specimens subjected to dynamic loads, the understanding of fatigue behavior is of great importance for avoiding unexpected material failure. The well-known S-N curve, also called the Wöhler curve, is typically used to determine the lifetime of materials, which is necessary for industrial design or material selection for specific applications. It shows the relationship between the applied load and the fatigue life of a material [1,2]. The S-N curve is typically separated into different ranges, which differ mainly in their fatigue damage processes. Conventionally, experiments in the Low Cycle Fatigue (LCF) regime are strain-controlled. The mathematical description is based on the approach of Manson and Coffin, which is given in Equation (1) [3,4,5]. The equation is composed of the following parameters: the plastic strain amplitude $\varepsilon_{a,p}$, the fatigue ductility coefficient $\varepsilon_{f}^{\prime }$, and the fatigue ductility exponent *c*.

(1) $$\varepsilon_{a,p}=\varepsilon_{f}^{\prime }\times {\left(2\times N_{f}\right)}^{c}.$$

In contrast to the LCF regime, experiments in the Very High Cycle Fatigue (VHCF) regime are mainly carried out in a stress-controlled manner. Due to the low stress amplitudes, a purely elastic description of the fatigue behavior according to Basquin is assumed [6]. With the stress amplitude $\sigma_{a}$, the Basquin coefficient *B* and the fatigue strength exponent *b*, Equation (2) can be obtained.

(2) $$\sigma_{a}=B\times {\left(2\times N_{f}\right)}^{b}.$$

The High Cycle Fatigue (HCF) regime of an S-N curve is characterized by an elastic-plastic material behavior. Therefore, a superimposition of the approaches according to Basquin (Equation (2)) and the Manson–Coffin (Equation (1)) is used in order to describe the S-N curve. The resulting Manson–Coffin–Basquin approach is given in Equation (3) [7].

(3) $$\varepsilon_{a,t}=\varepsilon_{a,e}+\varepsilon_{a,p}=\frac{\sigma_{f}^{\prime }}{E}\times {\left(2\times N_{f}\right)}^{b}+\varepsilon_{f}^{\prime }\times {\left(2\times N_{f}\right)}^{c}.$$

In Equation (3), $\varepsilon_{a,t}$ designates the total strain amplitude, whereas $\varepsilon_{a,e}$ is the elastic strain amplitude. A variety of approaches already exist for the determination of the required parameters of Equation (3). In 1965, Manson developed the four-point correlation method. It uses four different points on the elastic and plastic curves of the strain number of cycles plot. Each point is determined from tensile test data [8,9]. Based on this approach, a variety of other calculation methods have been developed which determine the parameters in Equation (3) [8,10,11,12,13]. Furthermore, there are calculation models that take the hardness of the materials, instead of the tensile strength, into account. As an example, the method according to Roessle [14] can be considered here. Moreover, energy-based models are used to describe the fatigue behavior of a material. In contrast to a correlation of the fatigue life with the stress or strain, these approaches have the aim of correlating the fatigue life with the plastic work performed per cycle. In order to describe the damage during the fatigue process, Ellyin and co-workers introduced a special form of the cyclic strain density as a suitable parameter. The total strain energy density $\Delta W_{t}$ results from the sum of the plastic portion of the strain energy density $\Delta W_{p}$ and the elastic portion $\Delta W_{e}$ [15,16,17,18].

(4) $$\Delta W_{t}=\Delta W_{e}+\Delta W_{p}.$$

The advantage of these methods is that the fatigue behavior can be described in the LCF as well as in the HCF regimes. For the reasons mentioned above, certain parallels can be drawn between the energy-based approaches and the approaches presented in this paper. Due to the thermal conductivity of metallic materials and the fact that nearly all the energy introduced is dissipated in terms of heat (approximately 90–95%), strain and temperature measurements correlate with each other. Similar to the energy-based approaches, the modifications of the StressLife method also attempt to obtain an accurate description of the S-N curve in the different regimes.

The goal of the newly-developed short-time procedure $StressLife_{HCF}$ (HCF = High Cycle Fatigue) is to enable a stress-controlled elastic-plastic description of an S-N curve in the HCF regime with as few specimens as possible. Conventional methods use a large number of specimens, which makes the generation of an S-N curve a very time- and cost-consuming procedure. In comparison, the developed method $StressLife_{HCF}$ requires only four fatigue tests to calculate all parameters of the S-N curve. The experiments consist of two load increase tests (LIT) and two constant amplitude tests (CAT). An advantage of the new method compared to the previously mentioned methods is that nondestructive measurement techniques (NDT) are used to detect the material response to dynamic loading. The values determined from this are directly included in the lifetime evaluation. This process-oriented view of the fatigue process instead of a lifetime-oriented view can increase the information content significantly. The $StressLife_{HCF}$ method evolved from the existing short-time method $StressLife_{tc}$ (tc = trend curve), whose explanation can be found in [19]. In addition to the procedure $StressLife_{HCF}$, the two variations $StressLife_{LHC}$ (LHC = Low and High Cycles) and $StressLife_{HVC}$ (HVC = High and Very High Cycles) were developed within the framework of the current research. The aim of these methods was to improve the representation of the transition areas of the S-N curve. The department of materials science and materials testing at the University of Applied Sciences Kaiserslautern has already developed a number of short-time procedures over recent years, such as SteBLife, StrainLife, and MiDAc-Life, which can be found in [20,21,22,23,24]. The StressLife method described in this paper differs from the other methods because the fatigue properties are described by an elastic-plastic approach, whereas SteBLife and MiDAc-Life are only based on an elastic and linear approach, respectively. The difference to StrainLife is that the experiments were performed under stress-controlled conditions instead of strain-controlled conditions. Furthermore, the StressLife method is clearly distinguished from previous methods by the calculation of the S-N curve in the transition regions.

## 2. Materials and Methods

### 2.1. Materials

The main focus within this research is the 20MnMoNi5-5 steel (1.6310), which is widely used for spraylines in German nuclear power plants. The steel is mostly comparable to the SAE 5120 considering the chemical composition. To demonstrate the validity of the $StressLife_{HCF}$ procedure, tests were additionally performed on the steel SAE 1045 (1.1191). The chemical composition of both materials can be seen in Table 1.

**Figure 1:**
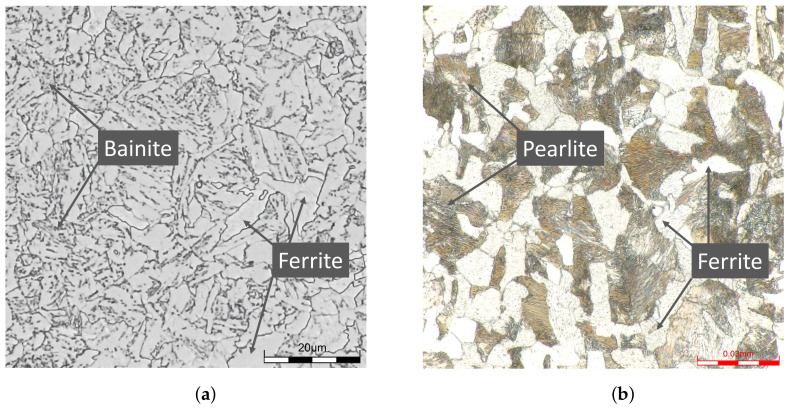
Micrographs of the steel 20MnMoNi5-5 and SAE 1045. (**a**) Micrograph of a 20MnMoNi5-5 steel in a quenched-tempered condition; (**b**) Micrograph of an SAE 1045 steel in a normalized condition.

The applied 20MnMoNi5-5 steel has a ferritic-bainitic microstructure, whereas the SAE 1045 has a ferritic-pearlitic structure. Micrographs of both materials, which were taken using a digital microscope type DSX1000 by the company EVIDENT (EVIDENT Europe, Hamburg, Germany), are given in Figure 1.

**Figure 2:**
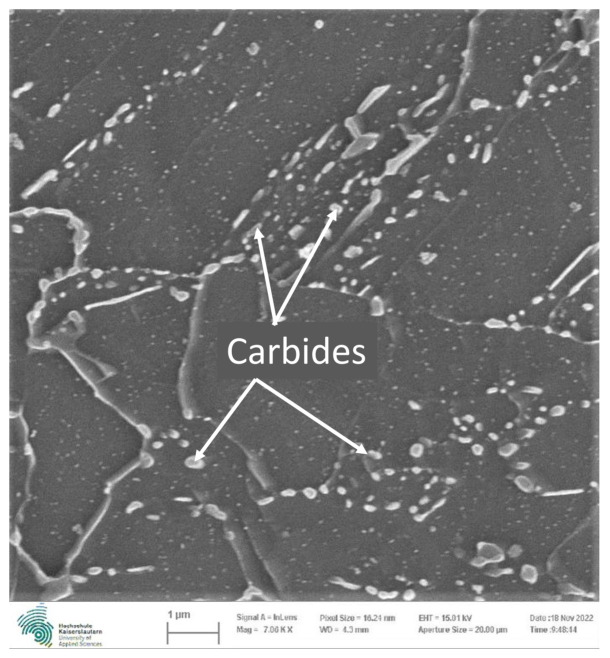
Microstructure of the 20MnMoNi5-5 steel including the carbides.

The ferritic-bainitic structure of the 20MnMoNi5-5 steel can be identified in Figure 1a. It results from a water-spray quenching of the pipes from which the specimens of the 20MnMoNi5-5 were taken. The microstructure consists of lighter areas, which represent the ferritic portion, and darker areas, which show the bainitic portion. The difference between the bainitic and the pearlitic structures can be derived very nicely from Figure 1a,b. While the bainitic structure is irregular and rather needle-shaped, the pearlite has a clear structure arranged in lamellae. The lighter areas in Figure 1b represent the ferrite analogue to Figure 1a. According to [25,26], carbides usually form as an additional phase in steels with a bainitic structure. Taking the limited diffusion possibilities of substitutional soluted atoms into account, it is assumed that most of the carbides are present as cementite $Fe_{3}C$. Due to the alloy composition of the material, additional Mn and Mo carbides are assumed. For an improved resolution of the carbides, a SEM image was taken using a GEMINI by ZEISS (Carl Zeiss, Oberkochen, Germany). The microstructure including the carbides can be seen in Figure 2.

### 2.2. Methods

**Figure 3:**
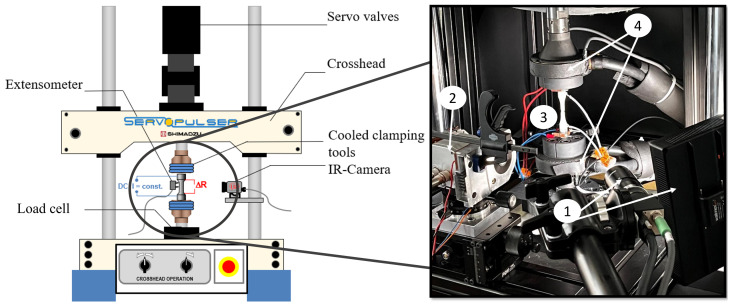
Experimental setup servohydraulic testing rig (EHF-L 20kN). (1) DIC system and illumination; (2) IR-camera thermoIMAGER TIM 450; (3) Electrical resistance measurement; (4) Watercooled clamps.

All fatigue tests (LIT as well as CAT) were performed at a testing frequency of 5 Hz at ambient temperature. The testing rigs are servo-hydraulic testing systems type EHF-L and EHF-U, with a maximum load capacity of 20 kN and 50 kN by the company Shimadzu (Shimadzu Europe, Duisburg, Germany). Both LIT and CAT were carried out stress controlled at a load ratio of R = −1, using a sinusoidal load-time-function. For the measurement of the cyclic deformation curves of the materials, different NDT-methods were used. The main focus was on the thermographic evaluations which operate with an IR-camera of the type TIM450 by Micro-Epsilon (Micro-Epsilon Messtechnik GmbH & Co. KG, Ortenburg, Germany) within the experiment. Furthermore, the strain was determined by means of a tactile extensometer and on selected tests by the use of a DIC-system by LIMESS (LIMESS Messtechnik und Software GmbH, Krefeld, Germany). The whole setup is given in Figure 3.

To record information in terms of the change in temperature, the specimen’s surface temperature was measured in three sections. $T_{1}$ is the temperature in the middle of the gauge length, whereas $T_{2}$ and $T_{3}$ transmit the temperature information at each shaft of the specimen. Because the deformations in the shafts are purely elastic due to the significantly larger diameter, Equation (5) can be used to describe the material response to cyclic loading based on the change in temperature ΔT.

(5) $$\Delta T=T_{1}-0.5\times \left(T_{2}+T_{3}\right).$$

The information from the change in temperature has great importance because it is closely linked to microstructural changes due to cyclically induced deformations. The theoretical background of the temperature measurement can be deduced from the consideration of the stress-strain hysteresis loop. The area of the hysteresis loop describes the cyclic plastic deformation energy, which is transformed into internal energy U and heat energy Q. The internal energy enables microstructural changes in dislocation structure and density, voids and pores, and micro- up to macro-cracks [27]. The temperature measurement is ideally suitable for the characterization of the material response to cyclic loading because of the good heat conduction properties of metallic materials. The predominant portion of 90% to 95% [28] of the plastic deformation work dissipates as heat and thus leads to an increase in specimen temperature. The remaining part is converted as internal energy, which is the cause of microstructural changes (e.g., dislocation reactions, micro- and macro-crack formation and propagation).

### 2.3. Fatigue Life Calculation Method StressLife_HCF_

$StressLife_{HCF}$ provides the basis for the complete evaluation process of the S-N curve, whereby the procedure is basically a modification of the original $StressLife_{tc}$-method. In order to generate a trend S-N curve with this method, only four specimens were required. As a first step, a LIT needs to be performed. The starting stress amplitude of the LIT must be well below the fatigue strength of the material. In general, the parameters for load increase tests can be estimated along the following criteria. The empirical relationship applicable here was developed on the basis of numerous results from completed investigations at the Department of Material Science and Materials Testing and validated on various unalloyed and low-alloyed steels (steels without phase transformation processes). Here, both the yield strength ratio and the ratio between the fatigue strength and yield strength (both are strongly dependent on the respective material condition) were included and correlated via the hardness (Vickers) with a correlation factor Z. For ductile materials, Z is 1.56 $\mathrm{MPa}\times \mathrm{HV}30^{-1}$, for medium strength materials 1.44 $\mathrm{MPa}\times \mathrm{HV}30^{-1}$ and, for high-strength materials, $\mathrm{MPa}\times \mathrm{HV}30^{-1}$.

(6) $$\sigma_{a,start,max}=HV\times Z-150\;\mathrm{MPa}.$$

**Figure 4:**
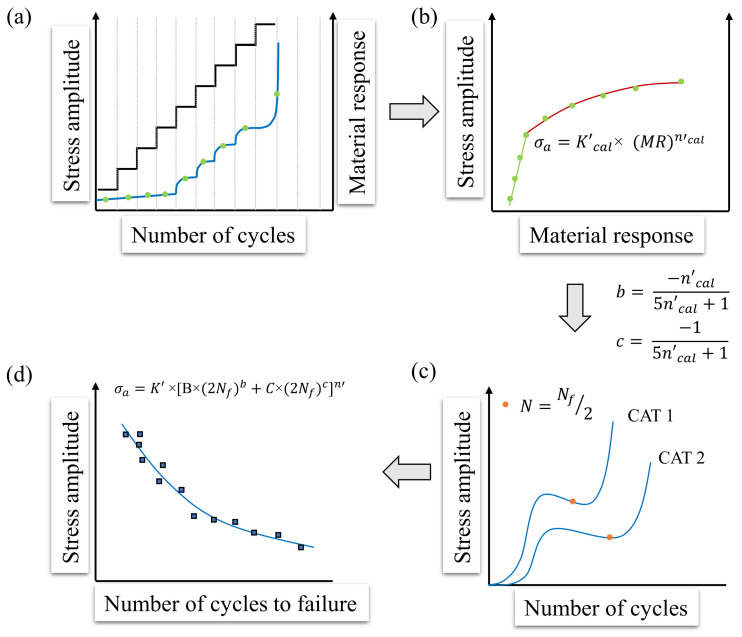
Schematic representation of the $StressLife_{HCF}$ method for calculating a trend S-N curve in the HCF regime with four specimens. (**a**) Slope of the stress amplitude and the material response of a LIT; (**b**) Stress amplitude of each load step plotted against the average value of the material response; (**c**) Cyclic deformation curve of both required CAT including the materials response at half of the cycles to failure; (**d**) S-N curve according to $StressLife_{HCF}$ including CAT data points for validation.

The resulting starting stress amplitude $\sigma_{a,start,max}$ indicates the respective maximum value (but may also be lower). In order to ensure a good comparability, most LITs were carried out with $\sigma_{a,start}=100$ MPa, in the case that the materials fulfil the criteria mentioned above. With regard to the step length in the LIT (at 5 Hz), 6000 load cycles for pure and predominantly softening materials, 9000 load cycles for alternating cyclic softening and hardening (predominantly ductile material states) have proven to be appropriate, since energetically stable states (especially with regard to the dissipated energy) occur within these cycle ranges. During this experiment, the material response on the cyclic loading as well as the step-wise load increase were measured in terms of the applied measurement techniques (thermography, resistometry, DIC). The original $StressLife_{tc}$-method only uses one LIT. Here, the problem arises in particular with brittle material behavior, as the generated data points in the plastic range are evidently too low and therefore an evaluation can only be carried out inadequately. For this reason, two LITs were used in the $StressLife_{HCF}$ method. The following boundary conditions for the two LITs must be taken into account: $\sigma_{a,start,2}>\sigma_{a,start,1}$ and $\Delta \sigma_{1}>\Delta \sigma_{2}$. This adjustment ensures an improvement in the quality of the final evaluation, especially in the elastic-plastic range. The measured cyclic deformation curve from the two LITs can be used as a first estimation of the fatigue strength of the examined material and to assess the appropriate stress amplitudes for the CATs [29,30]. The procedure for the determination of a trend S-N curve in the HCF regime according to the $StressLife_{HCF}$ method is given in Figure 4. A distinction was made between the cyclic hardening exponent $n_{cal}^{\prime }$ and the cyclic hardening coefficient $K_{cal}^{\prime }$, which are only used for the calculation of the parameters *b* and *c*, and the parameters $n^{\prime }$ and $K^{\prime }$, which are used in order to describe the S-N curve.

Figure 4a shows that the material response increases in the elastic-plastic range above a certain stress amplitude. Therefore, the fatigue strength can be estimated as the stress amplitude at which no material response occurs at last. In order to calculate the fatigue strength exponent *b* and the ductility exponent *c*, the average values of the material response of each load step were plotted against the corresponding stress amplitude, which can be seen in Figure 4b. It should be noted here that the use of the mean value is only permissible since there is no extensive cyclic hardening within the load steps. However, this relation is strongly influenced by the materials’ condition. The more brittle the material behavior is, the less pronounced the plastic range is. Being different from $StressLife_{tc}$, only the elastic-plastic behavior within the generalized Morrow plot was considered for the HCF regime. In order to take the material condition into account in the calculation of the fitting parameter $n_{cal}^{\prime }$, a weighting of the values for the elastic and the plastic portions was made. For this purpose, the elastic range, which, according to the authors, is defined as the last five elastic data points, and was fitted using an allometric fit. The plastic range was fitted in an analogue way. At this point, the value $\alpha_{pl}$ was introduced for the quantitative assessment of the plastic range. It shows the amount of data points in the plastic region of an LIT. With the slope of the elastic ($n_{el}$) and the plastic range ($n_{pl}$), the hardening exponent $n_{total}$ for the HCF regime can be determined according to Equation (7).

(7) $$n_{total}=\frac{5}{\alpha_{pl}+5}\times n_{el}+\frac{\alpha_{pl}}{\alpha_{pl}+5}\times n_{pl}.$$

According to Morrow [31], the weighted hardening exponent $n_{total}$ from Equation (7) can be used to calculate the required parameters from Equations (8) and (9).

(8) $$b=\frac{-n_{total}}{5\times n_{total}+1}$$

(9) $$c=\frac{-1}{5\times n_{total}+1}.$$

In the case of Equations (8) and (9), it must be noted that these are empirical relations and are therefore subject to error. However, due to the good agreement of the data of previous research, this influence was neglected. The specification of only five data points in the elastic range was necessary, as the evaluation would otherwise be dependent on the start amplitude of the LIT. Figure 4c shows an example of what cyclic deformation curves of CATs can look like (in the case of normalized ferritic-pearlitic steels). The slope starts with an increase of the material response, corresponding to a cyclic softening of the material due to dislocation reactions. A distinction can be made between dislocation movement, the formation of dislocation walls and cells, as well as changes in the dislocation density. The cyclic softening process was followed by a cyclic hardening process which is caused by the mutual obstruction of the dislocations, until the material finally showed secondary cyclic softening as a consequence of macroscopic crack formation and propagation. An advantage of calculating the S-N curve on the basis of the CATs is that transient cyclic effects in the plastic regime of the LIT can be compensated, thus ensuring high accuracy of the results. Due to the proven correlation between the plastic strain amplitude and the change in temperature, the material response can be separated into two portions in analogy to the strain-life approach [32]. The total response $M_{t}$ is given by the summation of the elastic ($M_{el}$) and the plastic portions ($M_{pl}$) [33].

(10a) $$\begin{array}{c} \;\;\;\;\;\;M_{t}=M_{el}+M_{pl} \end{array}$$

(10b) $$\begin{array}{c} M_{t}=\frac{\sigma_{f}^{\prime }}{E}\times {\left(2N_{f}\right)}^{b}+\varepsilon_{f}^{\prime }\times {\left(2N_{f}\right)}^{c} \end{array}$$

(10c) $$\begin{array}{c} M_{t}=B\times {\left(2N_{f}\right)}^{b}+C\times {\left(2N_{f}\right)}^{c}. \end{array}$$

The elastic content of Equation (10a) can be described by the generalized Basquin equation, whereas the plastic content is described by the Manson–Coffin equation. To determine the two coefficients B and C, Equation (10c) must be applied to both CATs. The material reaction needs to be tapped at a defined stage. Within this research, the definition is $N=0.5\times N_{f}$. By rearranging Equation (10c), Equations (11) and (12) can be obtained in order to calculate the missing parameters.

(11) $$C=\frac{{\left(2N_{f,1}\right)}^{b}\times \Delta T_{2}-{\left(2N_{f,2}\right)}^{b}\times \Delta T_{1}}{{\left(2N_{f,2}\right)}^{c}\times {\left(2N_{f,1}\right)}^{b}-{\left(2N_{f,1}\right)}^{c}\times {\left(2N_{f,2}\right)}^{b}}$$

(12) $$B=\frac{\Delta T_{1}-C\times {\left(2N_{f,1}\right)}^{c}}{{\left(2N_{f,1}\right)}^{b}}.$$

Based on this method, all parameters for the generation of a trend S-N curve (Figure 4d) in the HCF regime can be determined. The final expression used to describe the S-N curve is given by Equation (13).

(13) $$\sigma_{a}=K^{\prime }\times {\left[B\times {\left(2N_{f}\right)}^{b}+C\times {\left(2N_{f}\right)}^{c}\right]}^{n^{\prime }}.$$

### 2.4. Fatigue Life Calculation Method StressLife_LHC_

**Figure 5:**
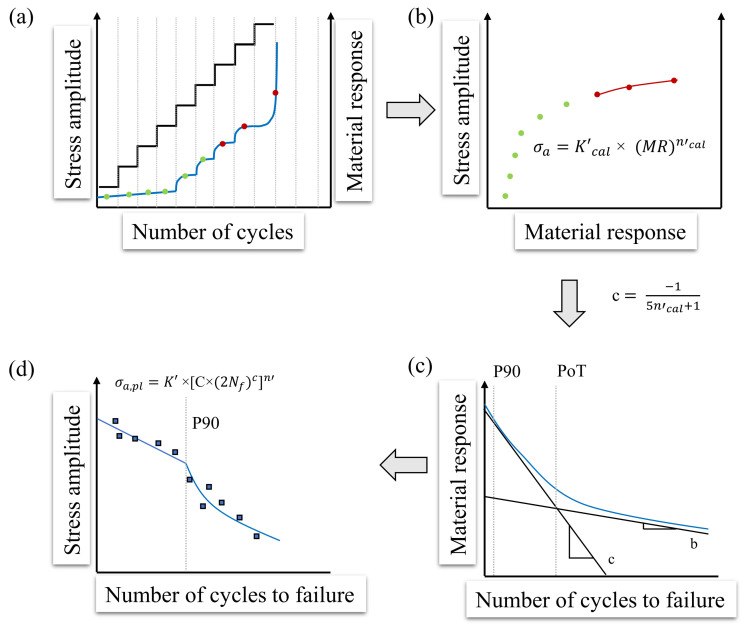
Schematic representation of the $StressLife_{LHC}$ method for calculating a trend S-N curve in the transition range to the LCF regime with four specimens. (**a**) Slope of the stress amplitude and the material response of a LIT; (**b**) Stress amplitude of each load step plotted against the average value of the material response; (**c**) Elastic, plastic and total material response plotted against the number of cycles to failure including the PoT and P90; (**d**) S-N curve according to $StressLife_{LHC}$ including CAT data points for validation in the transition range.

Based on the $StressLife_{HCF}$ method, further methods can be derived, which extend the description of the S-N curve beyond the HCF regime. In order to describe the S-N curve in the transition area to the LCF regime, $StressLife_{LHC}$ (LHC = Low and High Cycles) was developed. The schematic explanation of this method is given in Figure 5. It is assumed that the slope of the S-N curve must be different in the LCF and HCF regime due to different damage processes.

In this method, an LIT is carried out in an analogue procedure to $StressLife_{HCF}$ (Figure 5a). However, only data points from the plastic region are used in order to obtain the fitting data for the calculation of *c*, which is shown in Figure 5b. Because of the fact that in the transition to the LCF regime plastic deformation dominates, the elastic portion is neglected and therefore the fatigue strength exponent does not need to be calculated. Figure 5c shows the elastic, plastic, and total material response plotted against the number of cycles to failure. At this point, two definitions might be introduced. The point of transition (PoT) is defined as the number of cycles at which the elastic and the plastic portion of the material response are equal. In order to be able to define the point of the S-N curve at which the slope changes in the transition area, P90 is introduced. It is defined as the point at which the plastic portion accounts for 90% of the total material response. With the help of the anchor point from $StressLife_{HCF}$ at the P90 point and the known slope c, which is calculated by the fitting parameter from Figure 5b, the S-N curve in the transition area to the LCF regime can be identified. Equation (14) describes the S-N curve and takes only plastic parameters into account.

(14) $$\sigma_{a}=K^{\prime }\times {\left[C\times {\left(N_{f}\right)}^{c}\right]}^{n^{\prime }}.$$

### 2.5. Fatigue Life Calculation Method StressLife_HVC_

**Figure 6:**
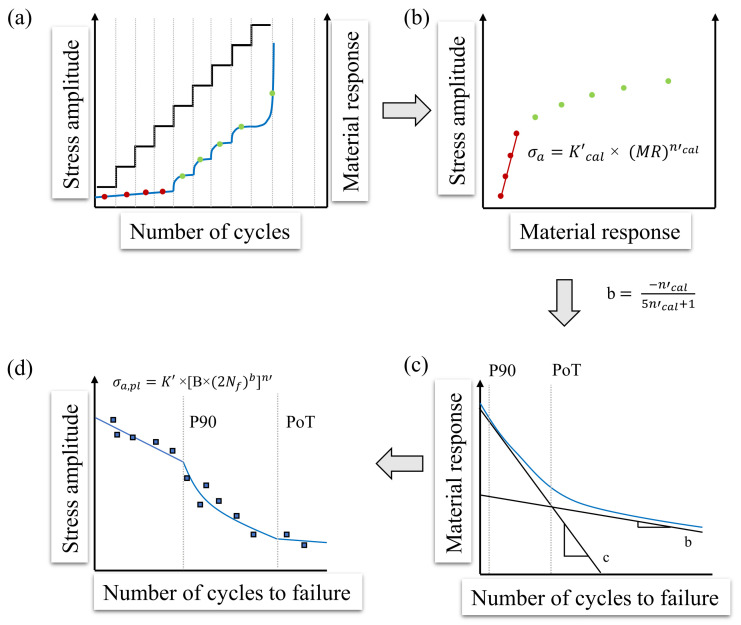
Schematic representation of the $StressLife_{HVC}$ method for calculating a trend S-N curve in the transition range to the VHCF regime with four specimens. (**a**) Slope of the stress amplitude and the material response of a LIT; (**b**) Stress amplitude of each load step plotted against the average value of the material response; (**c**) Elastic, plastic, and total material response plotted against the number of cycles to failure including the PoT and P90; (**d**) S-N curve according to $StressLife_{HVC}$ including CAT data points for validation in the transition range.

Contrary to the natural behavior of ferritic steels, the determined S-N curves according to $StressLife_{HCF}$ do not form a plateau at the transition to the VHCF regime. Because of this outcome, it is assumed that there must be another change of the slope when the elastic portion of the material response starts to dominate in comparison to the plastic portion. The approach of the $StressLife_{HVC}$ method (HVC = High and Very High Cycles) is to adopt a purely elastic description of the S-N curve, as the number of cycles of failure increases. A schematic overview of this method is given in Figure 6.

In contrast to the previously described methods, only the elastic data points from the LIT from Figure 6a were included in the calculation for the transition to the VHCF range. It is assumed that, in the VHCF regime, only damage processes with more elastic portions take place. For this reason, only the fatigue strength exponent b is required from the fitting parameters of Figure 6b. According to Wagener and Melz [34], the PoT should be indicated as a knee-point in the order of $10^{4}$ cycles. Below the PoT, the hysteresis loop shows a measurable plastic portion of the material response. Above this point, the plastic portion is assumed to be negligible. The elastic description of the S-N curve is given in Equation (15).

(15) $$\sigma_{a}=K^{\prime }\times {\left[B\times {\left(N_{f}\right)}^{b}\right]}^{n^{\prime }}.$$

## 3. Results and Discussion

### 3.1. Load Increase Test

**Figure 7:**
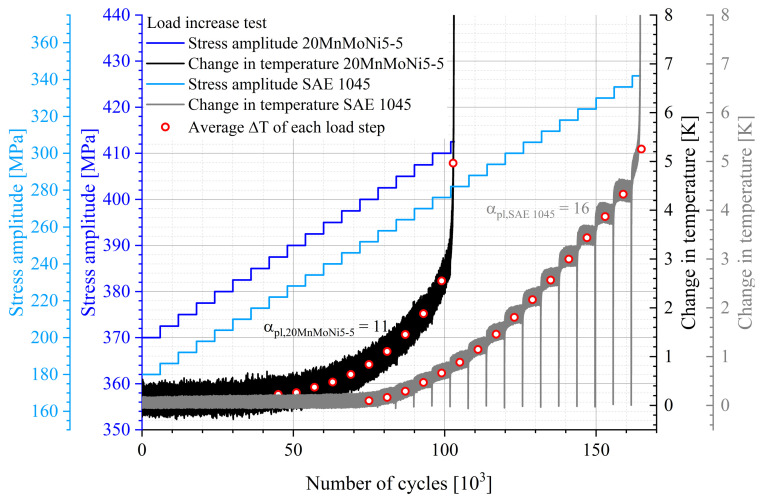
Cyclic deformation curve of a load increase test of the 20MnMoNi5-5 steel with $\sigma_{a,start}=370$ MPa, Δσ = 2.5 MPa and ΔN = $6\times 10^{3}$; Cyclic deformation curve of a load increase test of the SAE 1045 steel with $\sigma_{a,start}=180$ MPa, Δσ = 6 MPa and ΔN = $6\times 10^{3}$.

The basic requirement for the lifetime evaluation method according to $StressLife_{HCF}$ is always the performance of an LIT, which is given in Figure 7 for the two materials 20MnMoNi5-5 and SAE 1045. For simplification, the focus is on the presentation of the thermographically measured data of the second of the two LITs. It should be noted that other measurement techniques, such as electrical resistance or optical strain measurements, can also be used.

Figure 7 evidently reflects the different conditions of the two materials. The rather brittle behavior of the 20MnMoNi5-5 steel (black curve) leads to the result that only a small number of data points lie in the plastic range of the LIT, whereas the normalized SAE 1045 steel has a significantly higher portion of data points in the plastic range. The fact that the SAE 1045 steel exhibits a more pronounced material response than the 20MnMoNi5-5 steel can be explained at the microstructural level. Although the 20MnMoNi5-5 steel has a lower carbon content than the SAE 1045 steel, it has a higher number of alloying elements which lead to a lattice distortion and therefore less plastic deformation. Furthermore, the treatment condition of the material has a major influence on the cyclic deformation curve. The quenched-tempered condition of the 20MnMoNi5-5 leads to a higher dislocation density compared to the normalized condition of the SAE 1045. Materials with a higher dislocation density usually exhibit a pure cyclic softening. This softening process is expressed in the increase of the change in temperature in Figure 7. From the discussed reasons, it becomes evident that the evaluation according to StressLife depends on the material conditions. In order to take this into account, the value $\alpha_{pl}$ was introduced. It is a tool for the quantitative evaluation of the material behavior.

### 3.2. StressLife_HCF_

**Figure 8:**
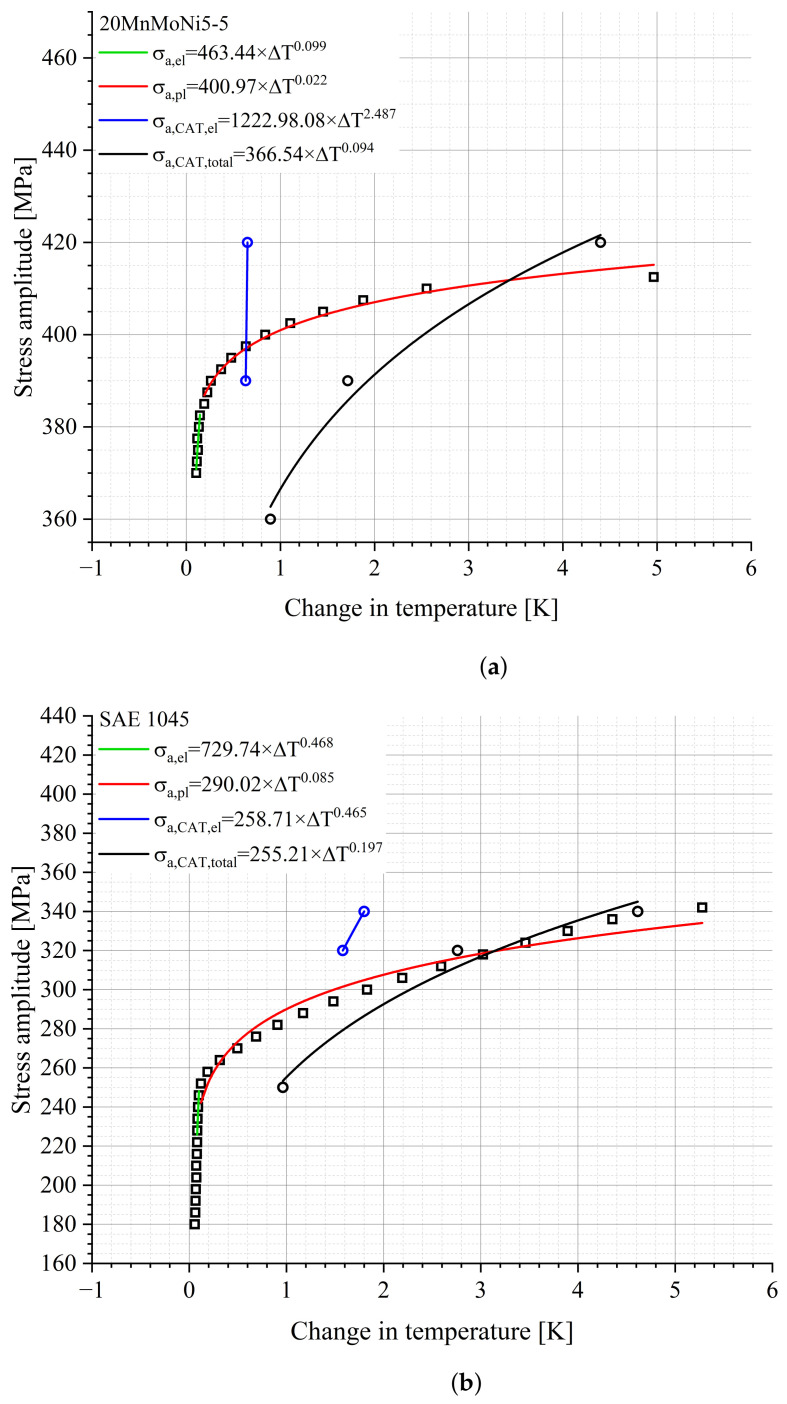
Relation between stress amplitude and change in temperature including the elastic change in the temperature of the CATs and the calculated CAT for $StressLife_{HCF}$ of (**a**) the 20MnMoNi5-5 steel and (**b**) the SAE 1045 steel.

The basic module of the presented new StressLife methods is $StressLife_{HCF}$. It was used to determine the S-N curve in the HCF regime. According to Figure 4, the relation of the stress amplitude and the material response can be derived from the LIT, which is shown in Figure 8.

Using the fit parameters from the allometric fit in the elastic and plastic ranges (Figure 8), the parameters n, b and c can be obtained according to Equations (7)–(9). The missing parameters B and C can be calculated from Equations (11) and (12). The calculated parameters are summarized in Table 2.

**Figure 9:**
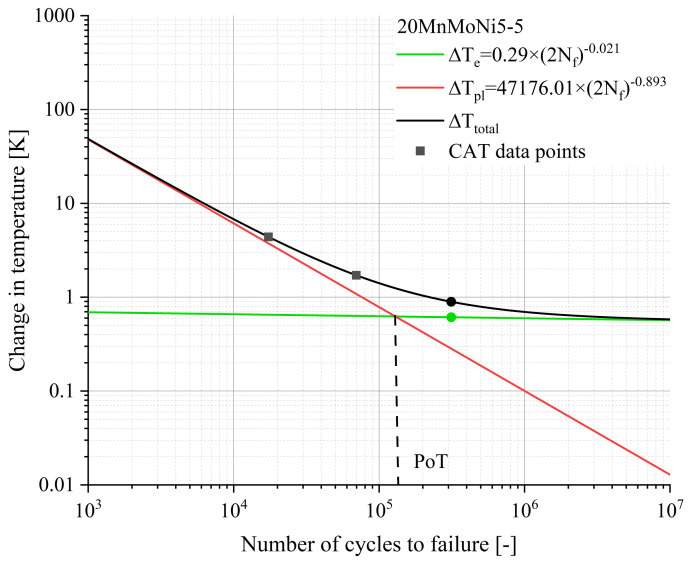
Calculated elastic, plastic, and total material response of the 20MnMoNi5-5 steel to an applied load as a function of $N_{f}$ according to Equation (10) with the experimental data derived from the CATs.

**Figure 10:**
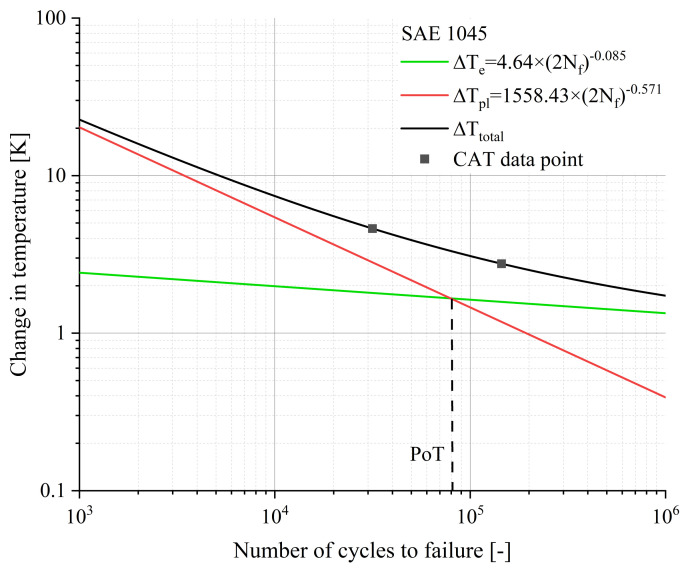
Calculated elastic, plastic, and total material response of the SAE 1045 to an applied load as a function of $N_{f}$ according to Equation (10) with the experimental data derived from the CATs.

According to Equation (10c), the material response is composed of an elastic portion, described by Basquin, and a plastic portion described by Manson–Coffin. Both, as well as the slope of the total material response, are given in Figure 9 and Figure 10.

The point marked as PoT (Point of Transition) in Figure 9 indicates the number of cycles at which the elastic and the plastic components of the material response have the same value. Above the PoT, the elastic portion of the material response is the predominant process, whereas below the PoT the plastic portion is the main process. From the Figure 9 and Figure 10, it can be deduced that the position of the PoT is highly material-dependent. Compared to the 20MnMoNi5-5 steel, the SAE 1045 shows a significantly higher material response and the intercept of the elastic function with the ordinate is about one order of magnitude higher than that of the 20MnMoNi5-5 steel. Consequently, the PoT shifts towards a lower number of cycles to failure.

**Figure 11:**
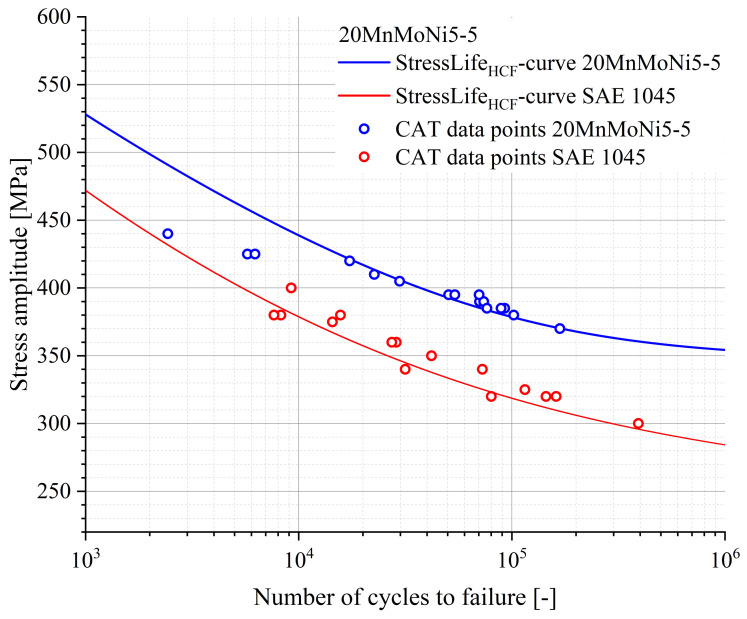
S-N curve according to $StressLife_{HCF}$ for a 20MnMoNi5-5 steel and an SAE 1045 steel, including CAT data points for validation.

To determine the S-N curve according to the $StressLife_{HCF}$ method, an additional CAT must be calculated. By using the data of this calculated CAT, the missing parameters of the StressLife curve can finally be determined. The determination of the missing parameters is the same as for $StressLife_{tc}$ and can be found in [19,35]. The final results for the calculation according to $StressLife_{HCF}$, including several CAT data points as a validation, are given in Figure 11.

Both curves are characterized by a high accuracy in the description of the CAT data points in the HCF regime. However, the SAE 1045 steel has a lower fatigue strength than the 20MnMoNi5-5 steel. This can be explained by the higher content of alloying elements in the 20MnMoNi5-5 steel. At this point, it must be taken into account that the slopes are based on the results of just one LIT. In order to be able to take the natural scatter of the materials into account, statistical evaluations need to be performed. Therefore, several LITs have to be carried out. By including the elastic and the plastic material responses in the $StressLife_{HCF}$ equation, the HCF regime can be described very well, but the transition to the adjacent regimes is not sufficient due to different damage processes. For this reason, two additional modules of the method were developed.

### 3.3. StressLife_LHC_

**Figure 12:**
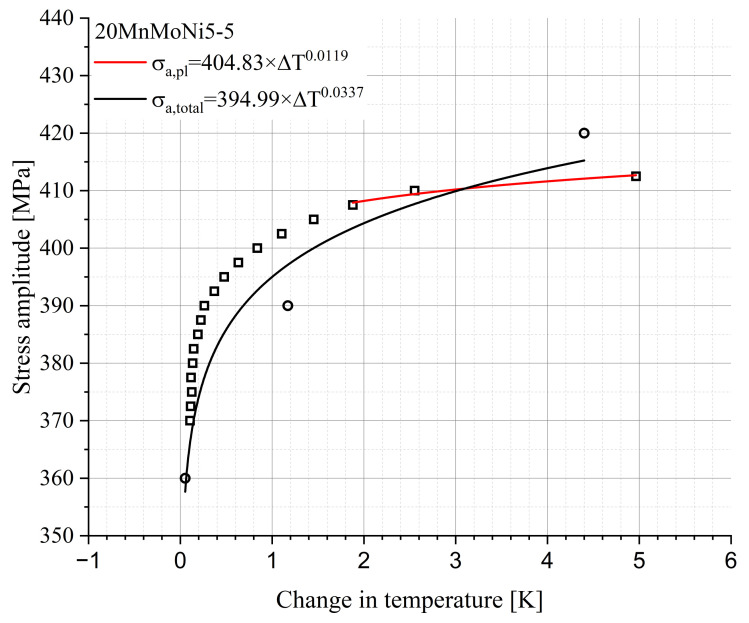
Relation between the stress amplitude and the change in temperature including the plastic change in the temperature of the CATs and the calculated CAT for the $StressLife_{LHC}$ calculation.

**Figure 13:**
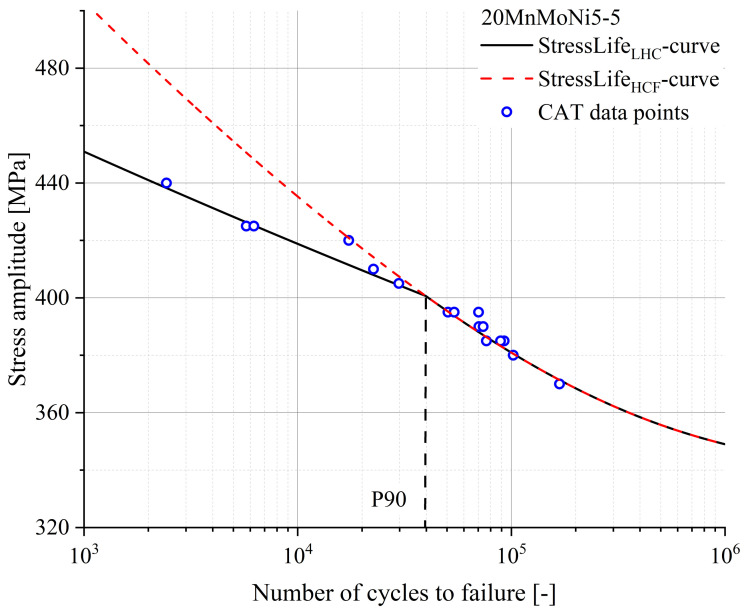
S-N curve according to $StressLife_{LHC}$ (black) and $StressLife_{HCF}$ (red) for a 20MnMoNi5-5 steel, including CAT data points for validation.

In the range of lower number of cycles to failure, there must be a change in the slope of the S-N curve due to changed damage processes. For example, the surface finish has a decisive influence on the HCF regime, while the surface loses influence in the range of lower cycle numbers. The CAT data points from Figure 11 of the 20MnMoNi5-5 steel, which lie below $10^{4}$ cycles, confirm this assumption. As described in Section 2.4, only the plastic data points of the Morrow equivalent plot were used, which can be seen in Figure 12. Compared to Figure 8, it is evident that the slope of the fit function is highly dependent on the selected data points. A consideration of only the plastic range leads to lower values of the slope than an elastic-plastic consideration. This leads to the consequence that parameters of the S-N curve change enormously. Using the Equations (9) and (14), the $StressLife_{LHC}$ curve can be determined. The result is given in Figure 13. Above the P90, the two curves of the $StressLife_{LHC}$ and $StressLife_{HCF}$ calculations are identical and describe the CAT data points with a high accuracy. Below the P90, the CAT data points are better described by the $StressLife_{LHC}$ curve, with one exception of a CAT at 420 MPa. A possible reason for the better accuracy of the $StressLife_{LHC}$ calculation is that the deformation in this fatigue range is mainly characterized through more plastic processes and thus the elastic-plastic description of the $StressLife_{HCF}$ cannot represent the realistic slope of the S-N curve. Table 3 summarizes all parameters of the $StressLife_{LHC}$ calculation.

Considering a transition point such as the one displayed in Figure 13, physical evidence also appears in these results, since without a change in the slope of the S-N curve, an extremely high intercept with the ordinate would be achieved.

### 3.4. StressLife_HVC_

**Figure 14:**
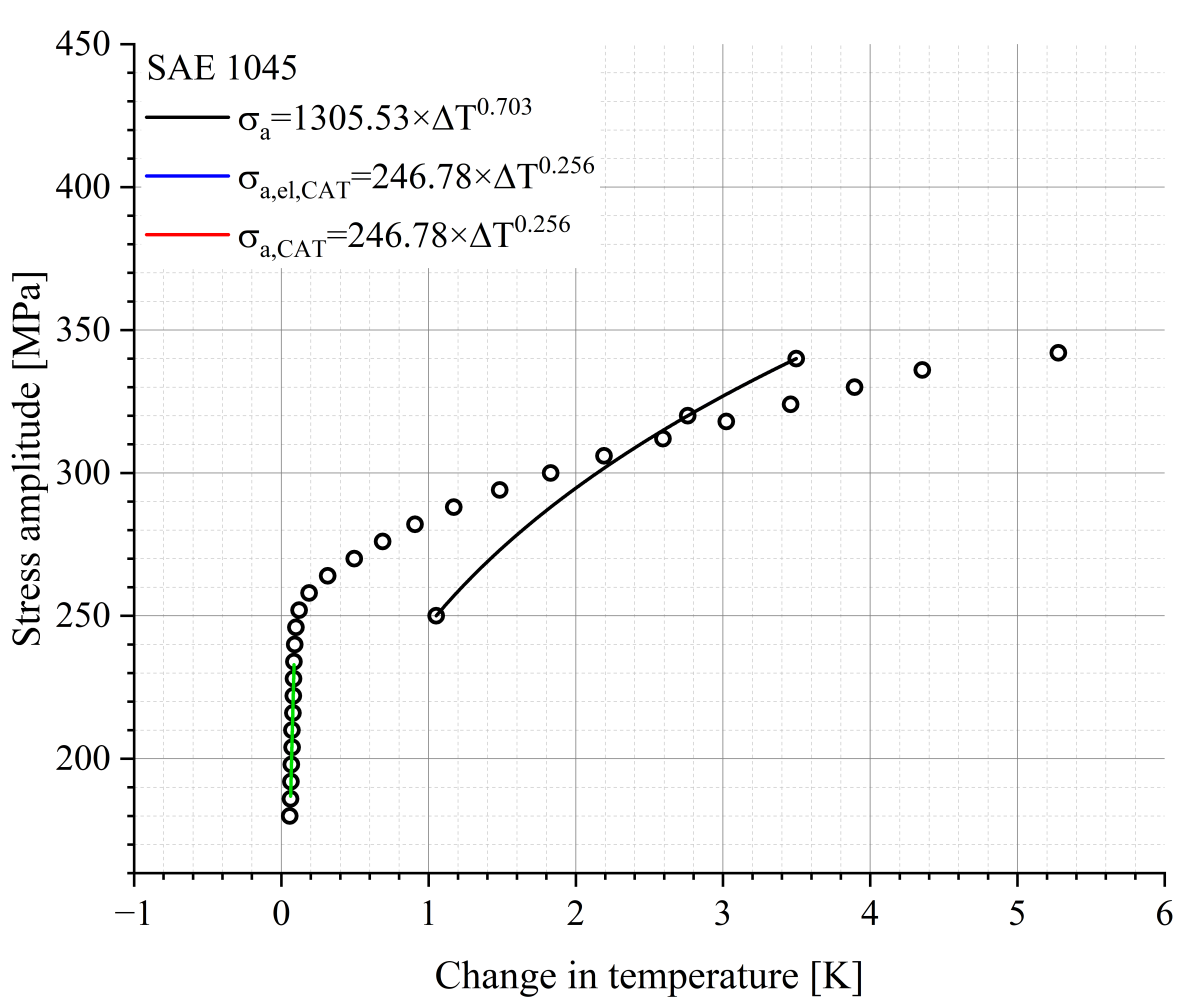
Relation between the stress amplitude and the change in temperature of an SAE 1045 including the elastic change in temperature of the CATs and the calculated CAT for the $StressLife_{HVC}$ calculation.

In the case of decreasing load amplitudes and, therefore, an increasing number of cycles, the elastic portion of the material response increases in comparison with the plastic portion. At the PoT, both parts of the material response have the same value. The position of the PoT is strongly material- and manufacturing dependent and can already occur in the HCF regime or at a higher number of cycles to failure in the transition to the VHCF regime. The approach of the $StressLife_{HVC}$ calculation is based on the assumption that only elastic data points of the Morrow-equivalent plot, which is given in Figure 14, are included in the calculation.

**Figure 15:**
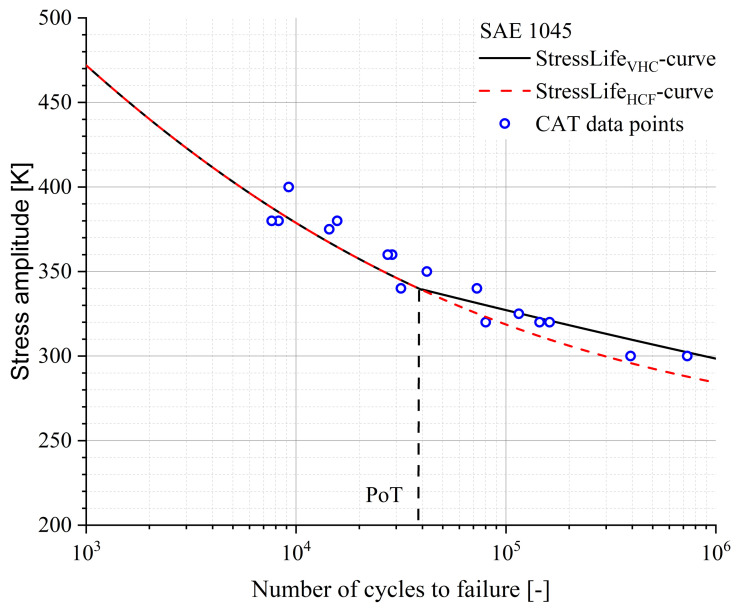
S-N curve according to $StressLife_{HVC}$ (black) and $StressLife_{HCF}$ (red) for a SAE 1045 steel, including CAT data points for validation.

A comparison of Figure 8b and Figure 14 provides the information that the slope used for the calculation is significantly larger in the case of the $StressLife_{HVC}$. This strongly influences the results of all other parameters. The evaluation was carried out in the same way as the evaluations of $StressLife_{HCF}$ and $StressLife_{LHC}$ with the exception that only the elastic parameters B and b were taken into account. The results of the lifetime evaluation according to $StressLife_{HVC}$ are given in Figure 15 and the parameters are summarized in Table 4.

Figure 15 shows that the $StressLife_{HCF}$ curve underestimates the CAT data points of the SAE 1045 in the area above the PoT. Compared to this, the $StressLife_{HVC}$ curve, which only takes the elastic slope of the Morrow-equivalent plot into account, describes the data points with a higher accuracy.

## 4. Conclusions

Within the scope of this research, the authors developed a new short time procedure designated as $StressLife_{HCF}$. The aim of this calculation method was to determine the lifetime of metallic materials with a small number of only four specimens. The method is based on the approaches according to Manson–Coffin for describing the plastic portion of the fatigue damage and Basquin in order to describe the process elastically. In addition, two modifications of the method have been developed, which are to be considered an expansion of the method. These methods can be summarized as follows:

### 4.1. StressLife_HCF_

- The method enables the generation of an S-N curve with a small specimen effort of a maximum of four specimens.
- By the combination of conventional fatigue testing methods and non-destructive measurement techniques, the information regarding ongoing fatigue processes is highly increased.
- The $StressLife_{HCF}$ equation consists of the approaches according to Manson–Coffin and Basquin.
- Through the extension to two load increase tests, the accuracy of the evaluation can be significantly increased.
- By weighting the elastic and plastic portions of the load increase test, the determination of the slope can be optimized.
- The generated S-N curve describes the constant amplitude test data points with a high accuracy.

### 4.2. StressLife_LHC_ and StressLife_HVC_

- Both methods are modifications of the $StressLife_{HCF}$ method, based on the same approaches and calculations.
- These modifications were developed in order to enable a description of an S-N curve beyond the high cycle fatigue regime.
- The relation of the material response and the number of cycles was used to calculate the anchor points PoT (Point of Transition) and P90 (90 % plastic part of the material response).
- The S-N curve was divided into three different ranges. At lower numbers of cycles, only the plastic part of the material response was considered in the calculation, whereas in the case of higher numbers of cycles only the elastic part was used.

The $StressLife_{HCF}$ process is to be further validated and expanded in additional tests, including with other materials such as pure metals, cast iron, and additive manufactured specimens. Furthermore, important influencing factors such as residual stresses and surface qualities are to be included in the method. The modifications $StressLife_{LHC}$ and $StressLife_{HVC}$, on the other hand, still need to be backed up with further tests in the respective areas. Another important aspect is the statistical validation via additional load increase tests and constant amplitude tests. Until now, only stress-controlled tests have been used for the developed procedure. In further research, strain-controlled tests should also follow.

## Figures and Tables

**Table 1:** Chemical composition in wt.−% of the 20MnMoNi5-5 and SAE 1045 steels according to our analysis and in comparison with ASTM A533B and DIN EN 10083-1.

Material		C	Si	Mn	Cr	Ni	Mo	S
1.6310	Certificate:	0.218	0.246	1.385	0.076	0.762	0.487	0.003
	ASTM:	0.190	0.200	1.290	0.120	0.800	0.530	0.008
1.1191	Certificate:	0.470	0.230	0.720	0.670	0.070	0.014	0.013
	DIN:	0.500	0.400	0.800	0.400	0.400	0.100	0.035

**Table 2:** Parameters from the $StressLife_{HCF}$ calculation.

Material	$n_{\mathrm{cal}}^{\prime }$ [-]	b [-]	c [-]	B [K]	C [K]	n′ [-]	K′ [$\mathrm{MPa}\times K^{-1}$]
**20MnMoNi5-5**	0.024	−0.021	−0.893	0.814	42,414.79	0.094	366.54
**SAE 1045**	0.15	−0.086	−0.571	4.642	1558.43	0.197	255.21

**Table 3:** Results of $StressLife_{LHC}$ calculation.

Material	$n_{\mathrm{cal}}^{\prime }$ [-]	b [-]	c [-]	B [K]	C [K]	n′ [-]	K′ [$\mathrm{MPa}\times K^{-1}$]
**20MnMoNi5-5**	0.012	-	−0.944	-	67047	0.034	394.40

**Table 4:** Results of the $StressLife_{HVC}$ calculation.

Material	$n_{\mathrm{cal}}^{\prime }$ [-]	b [-]	c [-]	B [K]	C [K]	n′ [-]	K′ [$\mathrm{MPa}\times K^{-1}$]
**SAE 1045**	0.703	−0.1557	-	20.13	-	0.256	246.78

## Data Availability

Data available on request.
